# Piezoelectricity of layered double hydroxides: perspectives regarding piezocatalysis and nanogenerators

**DOI:** 10.3762/bjnano.16.124

**Published:** 2025-10-20

**Authors:** Evgeniy S Seliverstov, Evgeniya A Tarasenko, Olga E Lebedeva

**Affiliations:** 1 Department of General Chemistry, Belgorod State National Research University, Pobedy 85, 308015 Belgorod, Russian Federation

**Keywords:** layered double hydroxides, nanogenerators, piezocatalysis, piezoelectricity

## Abstract

Recent research in alternate sources of energy such as piezoelectric energy conversion devices has positioned layered double hydroxides (LDHs) as promising candidates among the other two-dimensional materials. With their unique flexible layered structure, LDHs hold great potential for piezocatalysis and powering smart wearable electronics. Despite their promise, this area of study is still in its infancy and this review explores its recent advances. The discussion encompasses LDH-based piezoelectric nanogenerators, piezocatalytic and piezo-photocatalytic properties of LDHs, and composite material synergies that enhance the overall electroactive performance. Looking to the future, systematic research into the effects of LDHs’ composition and structure on piezoelectric properties will be crucial to unlock their full potential. This mini-review aims to inspire the audience with valuable ideas for the development of new LDH-based piezoelectric materials, thereby contributing to the development of next-generation high-performance piezoelectric devices.

## Introduction

Given the ever-increasing global need to reduce dependence on fossil fuels, finding other ways to generate energy from environmental sources is a priority. Piezoelectric materials have the ability to convert mechanical energy into electrical energy due to the arrangement of dipoles in their structure, thus providing sustainable electrical energy for low-power-consuming and self-powered devices [1].

The most common piezoelectric ceramics are lead-based, which can cause serious environmental damage if such compounds are widely used [2]. Among the new alternatives found in recent years is a group of layered double hydroxides (LDHs), whose piezoelectric properties were not previously known and systematically studied. Natural and synthetic layered hydroxides, also called hydrotalcite-like compounds, have a number of unique properties inherent to this class of materials [3]. LDHs easily undergo targeted modification because the composition of cations in the hydroxide layers and anions in the interlayer space can be varied over a wide range, allowing for the adjustment of key characteristics. Another specific feature of LDHs is their ability to delaminate in water and organic solvents after pretreatment to separate nanosheets. The latter is important in connection with the discovery of piezoelectric properties in two-dimensional materials, in some cases having an advantage over three-dimensional bulk counterparts [4]. Traditionally, LDHs are known as adsorbents, anion exchangers, catalysts, and catalyst precursors, but the wide range of their properties is not limited to this, as the discovery of piezoelectric properties has shown.

The aim of our work was to systematize the scattered current research on the piezoelectric properties of LDHs, for the first time providing an overall picture for current and future researchers in this and in related fields. Articles were searched using keywords that included variations of terms of the topics of piezoelectricity and layered double hydroxides in bibliographic systems and publishers such as ScienceDirect, Google Scholar, Royal Society of Chemistry, American Society of Chemistry, and MDPI. Our review is limited to articles published up to and including January 2025.

## Review

### Key findings and interpretations

#### Piezoelectric nanogenerators

**Figure 1:**
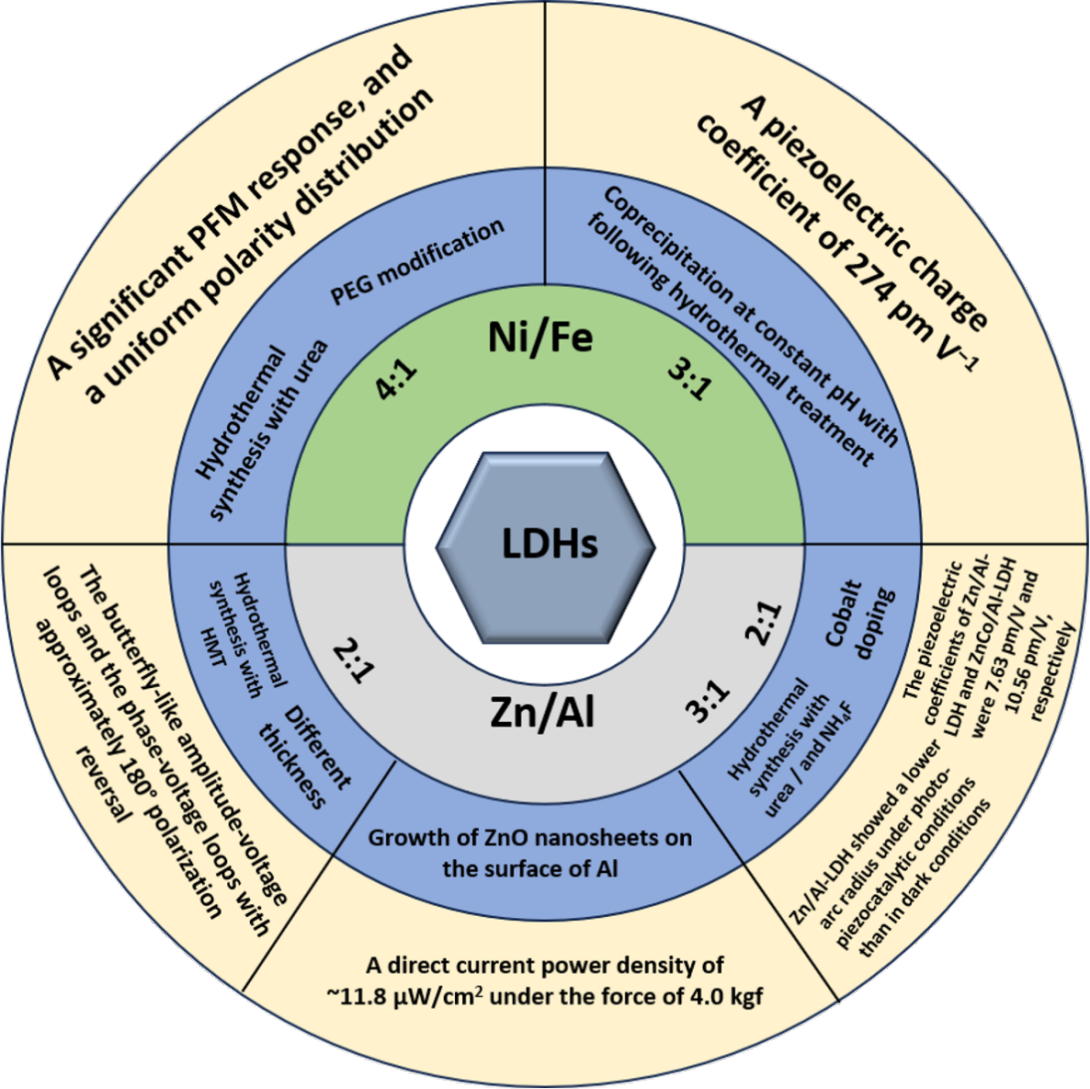
Visual summary of Table 1.

A general idea of the piezoelectric properties of LDHs in the described studies is given in Table 1. A visual summary of the described works is presented in Figure 1. Works where we assume the absence of a crystalline structure of LDHs despite the authors’ claims have been excluded from the table and the figure but left in the text.

**Table 1:** Overview of piezoelectric properties of LDHs.

LDH	Method of LDH synthesis	Method of piezoelectric properties studying	Piezoelectric properties	Ref.

PEG-modified Ni/Fe^3+^ (4:1)	hydrothermal with urea	piezoresponse force microscopy, ultrasonic current measurement, and electrochemical impedance spectroscopy	A significant PFM response was localized on the nanosheets, and a uniform polarity distribution was observed in phase mapping.	[5]
Zn/Al of different thickness (2:1)	hydrothermal with hexamethylenetetramine (HMT)	piezoresponse force microscopy, Kelvin probe force microscopy, transient current response measurement	When applying ± 80 V voltage on the surface of catalysts, butterfly-like amplitude–voltage loops and phase–voltage loops with approximately 180° polarization reversal were observed.	[6]
Ni/Fe^3+^-CO_3_ (3:1)	coprecipitation at constant pH with following hydrothermal treatment	piezoresponse force microscopy	A piezoelectric charge coefficient of 274 pm·V^−1^ was obtained.	[7]
Zn/Al-NO_3_	growth of ZnO nanosheets on the surface of Al via an aqueous solution route	a picoammeter and an oscilloscope for low-noise current and voltage measurements	A direct current power density of 11.8 μW/cm^2^ was achieved under the force of 4.0 kgf.	[8]
ZnCo^2+^/Al (2:1)	hydrothermal with urea and NH_4_F	piezoresponse force microscopy	The piezoelectric coefficients of Zn/Al-LDH and ZnCo/Al-LDH were calculated as 7.63 and 10.56 pm/V, respectively.	[9]
Zn/Al (3:1)	hydrothermal with urea	joint study of photo-piezocatalytic effect by transient photocurrent response and electrochemical impedance spectroscopy	Zn/Al-LDH showed a lower arc radius under photo-piezocatalytic conditions than in dark conditions, suggesting that it would accelerate the separation and fast transport of e^−^/h^+^ pairs	[10]

A Ni/Fe-LDH piezoelectric nanogenerator was used to charge an asymmetric supercapacitor made from the as-prepared Ni/Fe-LDH and biomass-derived activated carbon as the positive and negative electrode [7]. The Ni/Fe-LDH nanostructure itself demonstrated a piezoelectric charge coefficient of 274 pm·V^−1^. This work opens the door to future applications of LDHs in smart wearable electronic devices as both power generators and supercapacitors.

In addition to the intrinsic piezoelectric properties of LDHs, it has been demonstrated that LDHs, as anionic clays, play a crucial role in enabling direct current output from piezoelectric nanogenerators [8]. In particular, the abundant anions (NO_3_^−^) incorporated within the Zn/Al-LDH layer of the two-dimensional ZnO nanostructure are key contributors to achieving piezoelectric direct current output. The piezoelectric potential generated in the ZnO nanosheets functions as an applied voltage, facilitating charge storage within the LDH layers, which act as capacitors. This mechanism leads to enhanced voltage and current pulses under mechanical stimulation.

LDHs can also enhance the piezoelectric performance of such promising materials for energy harvesting as polyvinylidene fluoride (PVDF) through strong hydrogen bonding between the hydroxy groups of LDHs and the electronegative fluorine atoms of PVDF [11]. In the work of Shetty et al., incorporation of a low content of sodium dodecylbenzene sulfonate-modified Ni/Co-LDH as a filler promoted the formation of the electroactive β-phase in PVDF-based electrospun nanocomposite fabrics [12]. The enhanced piezoelectric response was attributed to the realignment of PVDF dipoles in combination with their interfacial interactions with the organically modified Ni/Co-LDH nanosheets.

However, some concerns arise regarding whether the synthesized material reported by the authors can indeed be classified as a Ni/Co-LDH. First, the synthesis was carried out using salts of divalent nickel and divalent cobalt at a Ni/Co ratio of 1:2. In the absence of trivalent cations, which are required to generate the excess positive charge necessary for stabilizing the LDHs structure, the formation of a pure LDH phase appears questionable. Moreover, the authors did not report any subsequent oxidation of nickel or cobalt to the trivalent state that could have compensated for this deficiency. Second, the presented XRD data are limited to 2θ values below 40°, whereas LDHs characterization is conventionally extended to at least 80° in order to capture the full set of reflections characteristic of the LDHs. Consequently, the data provided do not offer sufficient evidence to unambiguously confirm the formation of a Ni/Co-LDH phase.

#### Piezocatalysis

A large part of the published studies of piezoelectric properties of LDHs and combined materials based on them are focused not so much on the piezoelectric effect itself as on its application in piezocatalysis. This emerging area of catalysis utilizes the ability of piezoelectric materials to convert mechanical energy into electrical energy for subsequent electrochemical reactions [13].

Liu et al. suggested that ultrathin Ni/Fe-LDH modified with polyethylene glycol (PEG) for better water solubility and biocompatibility might exhibit a piezoelectric effect under ultrasound irradiation and generate superoxide anion radicals [5]. The results confirmed the potential of the selected LDH for application in novel piezocatalytic tumor therapy as an effective sonosensitizer. The obtained data indicated that the ultrasound exposure broke the inversion symmetry of Ni/Fe-LDH, causing positive and negative polarization charges to arise. The migration of created charges in opposite directions under external mechanical force caused an intrinsic electric field. The authors also hypothesized that LDHs, as some 2D layered bulk materials with centrosymmetric properties, display piezoelectricity when reduced to a single or few layers due to the loss of their inversion centers.

An actively developing area of piezocatalysis is its application to enhance photocatalytic reactions, called piezo-photocatalysis. Zn/Al-LDHs with different thicknesses were investigated for the piezo-photocatalytic degradation of carbamazepine [6]. The ultrathin samples achieved an efficiency of 95.8% with a reaction rate constant of 0.148 min^−1^. This effect was attributed to the higher sensitivity of ultrathin LDH sheets to both light irradiation and external stress, leading to increased current generation and reactive oxygen species production. The authors proposed that the piezoelectric field of LDHs facilitates the separation of photo-induced electron–hole pairs and modulates band alignment, thereby synergistically enhancing the piezo-photocatalytic process.

Other work was dedicated to imidacloprid degradation by activated peroxydisulfate [10]. In this study, Zn/Al-LDH served as a piezo-photocatalyst for peroxydisulfate activation. The degradation ratio for imidacloprid reached 90.6% with a reaction rate constant of 0.021 min^−1^, which was 205 and 29 times higher than that of individual Zn/Al-LDHs and LDHs combined with peroxydisulfate without light and stress exposure, respectively. This significant enhancement was also attributed to the stimulation of a high current by the photo-piezocatalytic effect generating electrons and holes in Zn/Al-LDHs.

The crystal structure of LDHs is fairly flexible, enabling adjustment of their cationic composition. One widely used method for preparing LDH-based catalysts is cation doping further increasing their catalytic activity. A cobalt-doped Zn/Al-LDH (ZnCo/Al-LDH) piezoelectric catalyst was used for activating peroxymonosulfate (PMS) to degrade norfloxacin [9]. The obtained catalyst demonstrated effective degradation within 15 min, achieving a degradation efficiency of 91.50% and a rate constant of 0.1644 min^−1^. In this study, the main PMS activation mechanism was non-radical. The piezoelectric properties of the ZnCo/Al-LDH-catalyst accelerated carrier transport and promoted regeneration of the Co^2+^ catalytic active center.

Another variety of LDH-based catalysts are composite materials produced from constituent materials with LDHs as one of them. When combined with BaTiO_3_, Zn/Al-LDH demonstrated great piezo-photocatalytic capabilities, achieving degradation rates of 99% for nitenpyram and 100% for tetracycline hydrochloride within 45 min [14]. Notably, the degradation was performed with coexistent pollutants. These composites exhibited enhanced piezoelectric properties compared to Zn/Al-LDH alone. The authors proposed that the enhanced efficiency was due to the interaction between the lamellar structure of Zn/Al-LDH and the piezoelectric attributes of BaTiO_3_, along with heterojunctions within the BaTiO_3_/LDH composite. However, this study raises doubts concerning the successful formation of a crystalline LDH structure. First, the reported cation ratio of divalent (Zn^2+^) to trivalent (Al^3+^) species is approximately 0.3, which is inconsistent with the well-established requirement that divalent cations must predominate in order to stabilize the LDHs structure. Second, the presented XRD patterns lack the characteristic LDHs reflections, particularly the (003) and (006) peaks typically observed at 2θ values of around 10° and 20°, respectively. The absence of these reflections strongly suggests that the samples do not exhibit the structural features characteristic of LDHs.

In addition to zinc-aluminum LDHs, piezo-photocatalytic properties have also been studied for Ni/Fe LDH [15]. A Ni/Fe-LDH/Bi_2_MoO_6−_*_x_* composite has been designed for the piezo-photocatalytic N_2_ oxidation to NO_3_^−^. The obtained material displayed a high nitric acid production rate (7.23 mg·g^−1^·h^−1^). Experimental evidence demonstrated that dual oxygen vacancies in Ni/Fe-LDHs create interfacial bonds that mitigate carrier localization effects and unfavorable influences during piezocatalysis and photocatalysis. These features promote exciton dissociation and charge transfer. Furthermore, the strong electronic interactions within the interfacial bonds induced internal structural reconstruction, which enhanced local polarization and N_2_ adsorption. As a result, the cleavage of N≡N bonds was accelerated, and the activation energy of the reaction was reduced. Unfortunately, this study also raises concerns regarding the successful synthesis of a hydrotalcite-like Ni/Fe-LDH. The reported cation ratio of Ni/Fe = 0.56 is unlikely to yield a stable LDH phase. Moreover, the presented XRD data begin only at 2θ values of 20°, thereby omitting the (003) reflection at approximately 10°, which is one of the most critical indicators of layered structure. The remaining reflections are barely discernible, broad, and of low intensity, further suggesting that the obtained material is predominantly amorphous phase rather than crystalline LDH.

The piezocatalytic performance of the abovementioned LDHs is quite impressive in many cases (e.g., 95.8% degradation of carbamazepine, *k* ≈ 0.148 min^−1^ for ultrathin Zn/Al-LDH; 91.50% norfloxacin degradation, *k* ≈ 0.1644 min^−1^ for ZnCo/Al-LDH); also, it is useful to compare them with benchmark non-LDH piezocatalysts. For example, BaTiO_3_ nanomaterials, long considered a standard for piezocatalytic environmental remediation, achieved ≈98% degradation of rhodamine B within 20 min under ultrasonic activation when used together with PMS, with a kinetic constant significantly higher than many simple photocatalysts [16].

Thus, we can conclude that LDHs are competitive with, and in several cases even surpass, established individual piezocatalysts like BaTiO_3_ in terms of degradation efficiency or reaction rate, especially when LDHs are ultrathin or doped/composited. However, many BaTiO₃-based systems still offer advantages in stability, availability of well-studied defect engineering, and structural robustness. Therefore, the strength of LDH-based piezocatalysts lies especially in their tunability (composition, thickness, and interlayer anions) rather than in a maximum rate alone, making them promising alternatives or complements in piezocatalytic applications.

## Conclusion

The study of piezoelectric properties of layered double hydroxides is still at an early stage. Nevertheless, current results show that LDHs certainly have the potential to be further integrated into future piezoelectric devices, both as main energy generators and as auxiliary agents that enhance the electroactive properties of the base material. The main feature that distinguishes LDHs is their dual activity as both generators of their own piezoelectric field and charge-storing supercapacitors. Charge-balancing anions between the LDHs layers can also contribute to the overall electroactive performance.

Overall, the discussed studies appear largely consistent; ultrathin structures, doped systems, and composites systematically show improved performance in applications where mechanically induced charge generation and utilization are important (piezo-catalysis/piezo-photocatalysis, energy storage). Three interrelated parameters, reduced thickness, cationic flexibility, and heterostructure formation, emerge as recurring factors that enhance charge separation and local polarization. Thus, the idea that LDHs can act not only as passive hosts but also as active piezoelectric components is supported by all mentioned works.

At the same time, our review also reveals methodological and interpretative weaknesses that complicate direct comparison of results. These weaknesses are: (1) Lack of unified metrology: In many reports, piezoelectrical properties are derived from indirect effects (enhanced degradation under ultrasound or higher current under mechanical stress) rather than from standardized direct measurements (e.g., *d*_33_ coefficients, calibrated PFM, polarization–voltage loops, or macroscopic electromechanical tests). This makes it difficult to determine whether the observed effects are truly piezoelectric in origin, or instead arise from triboelectric, electrochemical, or mechanochemical contributions. (2) Confusion of mechanisms: Different studies attribute performance improvements either to symmetry breaking (ultrathin LDH, loss of inversion centers, piezoelectrical properties), to defects (oxygen vacancies and carrier localization effects), or to interfacial electronic interactions that facilitate charge transfer. While these mechanisms may coexist, many publications lack the necessary control experiments (e.g., mechanical stimulation without light/oxidant, photocatalysis without stress, or defect-free samples) to rigorously disentangle their respective contributions. As a result, similar activity enhancements are sometimes explained in fundamentally different ways. (3) Stability and reproducibility: For practical applications (energy harvesting, catalytic cycling, and device integration), long-term stability, reproducibility of synthesis (phase purity, secondary phases, and role of interlayer anions), and durability under repeated operation are critical.

From this perspective, several steps could strengthen the further subject studies: (1) reporting standardized, direct piezoelectric measurements (*d*_33_, calibrated PFM mapping, and macroscopic tests) alongside application metrics (rate constants and degradation efficiency); (2) systematically including control experiments to isolate different contributions; (3) evaluating phase purity and interlayer chemistry, given their influence on charge mobility and capacitance; and (4) assessing long-term stability and synthesis reproducibility.

Furthermore, at present, piezoelectric properties are being studied only for Zn/Al-LDHs or Ni/Fe-LDHs. Do other sets of divalent and trivalent cations or other sets of charge-balancing anions in LDHs exhibit similar properties? In one case, doping a binary LDH with third cation improved its electroactive performance. Which dopants would be most efficient for improving piezoelectric properties? Will new unexpected results emerge from multication LDHs?

Only by addressing abovementioned points and questions can this field move beyond promising case studies toward a complete understanding of when and why LDH-based systems truly outperform or complement conventional piezoelectric materials.

## Data Availability

Data sharing is not applicable as no new data was generated or analyzed in this study.
